# Undertriage of geriatric trauma patients in the emergency department: A retrospective single-center study

**DOI:** 10.1007/s40520-026-03465-6

**Published:** 2026-08-06

**Authors:** Małgorzata Sulej-Niemiec, Andrzej Kopta, Justyna Mazur, Monika Bednarek-Chałuda, Aleksandra Załustowicz, Paweł Kukla, Agnieszka Micek

**Affiliations:** 1 Faculty of Health Sciences, Department of Emergency Medical Services, Jagiellonian University Medical College, Michałowskiego St. 12, 31-126, Kraków, Poland; 2 Centre of Innovative Medical Education, Department of Medical Education, Jagiellonian University Medical College, Kraków, Poland; 3 Faculty of Medicine, Department of Orthopedics, Jagiellonian University Medical College, Kraków, Poland; 4 Faculty of Health Sciences, Institute of Nursing and Midwifery, Statistical Laboratory, Jagiellonian University Medical College, Kraków, Poland

**Keywords:** Geriatric trauma, Emergency department triage, Geriatric triage, Undertriage, Trauma triage, Head injuries

## Abstract

**Aim:**

To analyze emergency department (ED) triage outcomes in geriatric trauma patients and evaluate undertriage in relation to injury severity.

**Methods:**

A retrospective analysis was conducted on anonymized medical records of patients aged ≥ 65 years admitted to the ED of the University Hospital in Kraków, Poland, due to trauma between 2018 and 2020. Among 111,615 total ED admissions, 4,922 geriatric trauma cases were identified. Triage categories (adapted ESI) were evaluated in relation to the Injury Severity Score (ISS), Trauma and Injury Severity Score (TRISS), and Glasgow Coma Scale (GCS). Undertriage was defined as an ISS ≥ 16 with assignment to *lower-risk triage priorities (3–5).*

**Results:**

Undertriage occurred in 34.4% of severely injured geriatric patients, exceeding the recommended threshold of < 5%. Head and neck injuries were the most severe across triage categories (AIS 3–4). Among patients with head and neck injuries, those in *the undertriage group 65 +* had significantly higher GCS scores (median 15) than those in *the correct triage group 65+* (median 5), suggesting that preserved consciousness may mask severe injuries. Overtriage was observed in 37.6% of cases, frequently involving head trauma with high GCS scores.

**Conclusions:**

These findings highlight the need for age-specific approaches to ED triage, particularly in cases of head trauma. In this cohort, commonly used physiological triage parameters, including the GCS, were associated with limitations in identifying patients with more severe injuries. These findings support further research on geriatric-specific triage approaches that account for age-related factors potentially affecting triage accuracy and the management of older trauma patients.

## Background

Population aging, particularly in highly developed countries, represents a major demographic shift and an increasing challenge for healthcare systems due to the growing number of geriatric patients [1]. In Europe, this age group is expected to constitute nearly one-third of the population by 2050 [2]. At the same time, older adults remain increasingly active, and trauma-related hospitalizations in this age group continue to rise [3, 4].

Trauma in older adults is associated with high mortality, long-term disability, loss of independence, and an increased need for institutional care [2, 4, 5]. These outcomes are influenced not only by injury severity but also by age-related physiological changes, multimorbidity, and polypharmacy, all of which may complicate early assessment and management [1, 5, 6]. In particular, older patients may present with apparently stable vital signs despite serious injury, which increases the risk of undertriage [7, 11].

Some of these negative consequences may be mitigated by adapting trauma systems to specific characteristics of the geriatric patient group. Emergency departments (EDs) and trauma centers (TCs) must be prepared for the increasing number of older trauma patients, including the optimization of triage protocols [6].

Triage is applied at multiple stages of emergency care, ranging from prehospital triage to initial in-hospital assessment, while taking into account the patient’s current condition and known comorbidities [1]. Although the physiological response to trauma evolves over time, the initial vital signs recorded in the ED remain crucial for clinical assessment and therapeutic decision-making [6].

The management of geriatric trauma patients is inherently complex, and accurate ED triage is essential in geriatric trauma care; however, most triage systems were not specifically developed for this population, and their performance remains uncertain.

Previous studies have emphasized undertriage in older trauma patients, but most have focused on prehospital triage or trauma team activation (TTA) rather than routine ED triage. Moreover, data on how ED triage priority corresponds to actual injury severity in geriatric patients remain limited, particularly within the national trauma system. Improving care for this population is essential, as its quality constitutes an important indicator of trauma system maturity and effectiveness.

### Aim

To analyze emergency department (ED) triage outcomes in geriatric trauma patients and to evaluate the extent of undertriage in relation to injury severity.

## Materials and methods

A retrospective analysis was conducted using anonymized hospital medical records of patients admitted between 2018 and 2020 to the Emergency Department (ED) of the University Hospital in Kraków, Poland, which operates an accredited Trauma Center (TC). The ED has no formally defined catchment area. It is one of six emergency departments in Kraków and one of 21 currently operating in the Lesser Poland Voivodeship, receiving both self-presenting patients and those transported by emergency medical services according to the shortest estimated travel time. In contrast, as the only TC in the voivodeship and one of 17 in Poland, the hospital has a formally defined trauma care coverage area of at least 1 million inhabitants within a 1.5-hour transport radius.

Trauma-related ED admissions were identified using ICD-10 codes recorded in the hospital medical records. The screening included all ICD-10 diagnosis codes related to injury, including S00–S99, T00–T35, T79, and T81–T95. External-cause codes were also screened, including V01–V99, W00–W64, X00–X19, X33–X39, X70, X72–X82, X91, X93–X99, Y00–Y05, Y20, Y22–Y32, Y60–Y61, and Y85. Codes related to complications of medical and surgical care were considered only when the clinical documentation confirmed an acute traumatic presentation assessed in the ED. The ICD-10 code search was used only for case identification; final confirmation of trauma status and injury-severity classification were based on a detailed review of medical records and the calculation of ISS and TRISS.

### TRISS methodology

**The trauma and injury severity score (TRISS)** estimates the probability of survival (Ps), expressed as a percentage, by combining anatomical and physiological indicators of trauma severity using a logistic regression equation. The key components of the TRISS model include the Revised Trauma Score coding (RTSc), Injury Severity Score (ISS), type of injury, and an age factor, which is particularly relevant in geriatric patients [12, 13].

Revised Trauma Score coding (RTSc) is the physiological component of the TRISS model, assessing trauma severity based on physiological parameters. It evaluates respiratory rate (RR), systolic blood pressure (SBP), and the Glasgow Coma Scale (GCS) [22].

**The injury severity score (ISS)** constitutes the anatomical component of the TRISS model and quantifies the severity of multiple injuries across the following body regions: head and neck, face, chest, abdomen or pelvic contents, extremities or pelvic girdle, and external (including skin, subcutaneous tissue, and burns). The ISS is calculated as: **ISS** = (highest AIS score)^2^ + (second highest AIS score)^2^ + (third highest AIS score)^2^.

The American College of Surgeons Committee on Trauma (ACSCOT) defines severe injury as an ISS ≥ 16 and recommends that these high-risk trauma patients be treated in major trauma centers [14].

ISS scores categorize the severity of multiple injuries as follows: <9 mild, 9–15 moderate, 16–24 severe, and ≥ 25 profound [14, 15].

The Injury Severity Score (ISS) is based on the Abbreviated Injury Scale (AIS), which quantifies the severity of individual injuries using medical documentation, including imaging findings. Each injury is assigned a severity score on the AIS scale, ranging from 1 (minor) to 6 (maximal) [16].

Both ISS and TRISS are objective tools for the assessment of geriatric trauma patients and have been validated as predictors of mortality. However, because they rely on data that are only available retrospectively (e.g., the results of diagnostic procedures), they are not suitable for real-time triage. Nevertheless, they allow for a reliable analysis of distributions and relationships across triage groups, as well as comparison of outcomes with data from international studies, and they serve as objective tools for evaluating triage methods.

As no dedicated trauma registry or trauma registrar was available during the study period, ISS and TRISS were calculated retrospectively by a single researcher based on detailed review of patients’ medical records.

### Triage system in the emergency department (ED)

The triage system implemented in the analyzed ED was based on an adaptation of the Emergency Severity Index (ESI), which classifies patients into five levels of admission priority:

1. Immediate: life-saving intervention required.
2. High-risk: high-risk situation or unstable vital signs.
3. Expanded diagnostic and treatment resources required.
4. One diagnostic and treatment resource required.
5. No diagnostic or treatment resources required [17].

As part of a local protocol, the facility modified the triage scheme by introducing colour codes inspired by the Manchester Triage System [18].

This triage system was subsequently adopted as a nationwide standard; however, no detailed formal validation procedures were established. According to the Regulation of the Minister of Health, the following maximum waiting times in the ED were established for each urgency category:

1. **RED**: immediate contact with a physician.
2. **ORANGE**: up to 10 min.
3. **YELLOW**: up to 60 min.
4. **GREEN**: up to 120 min.
5. **BLUE**: up to 240 min [19].

Triage was performed immediately upon ED arrival by designated on-duty paramedics or nurses trained in the local adapted ESI-based triage protocol, regardless of whether patients arrived by ambulance or as walk-in patients. No formal assessment of inter-rater reliability was available in the retrospective documentation; therefore, potential variability between triage personnel should be considered when interpreting triage accuracy.

For the purposes of this analysis, the study population was categorized according to patient urgency:

- **high-risk priorities**: 1. RED + 2. ORANGE (1 + 2),
- **lower-risk priorities**: 3. YELLOW + 4. GREEN + 5. BLUE [3–5].

(Li et al., 2024; Zaboli et al., 2021; Nishi et al., 2018; Lukin et al., 2015) [18, 20].

**Undertriage** was defined as severe injury (ISS ≥ 16) assigned to **lower-risk priorities** [3–5].

**Overtriage** was defined as non-severe injury (ISS < 16) among patients assigned to **high-risk priorities (1 + 2)**.

These rates were used to compare ED urgency assignment with retrospectively determined anatomical injury severity.

### Statistical methods

Statistical analyses and graphical visualizations were performed using R: A Language and Environment for Statistical Computing (R Core Team, version 4.4.1). Normality of quantitative variables was assessed using the Shapiro–Wilk test, skewness and kurtosis measures, and visual inspection of histograms. Descriptive statistics were presented as means with standard deviations (SD) or medians and interquartile ranges (IQR), as appropriate depending on data distribution. Qualitative variables were summarized as counts and percentages. Appropriate statistical tests, including the Mann–Whitney U test, Student’s t-test, and chi-square test, were applied for comparisons between analyzed variables. A multivariable logistic regression model was used to identify characteristics associated with undertriage. In all analyses, a significance level of α = 0.05 was used.

## Results

**Fig. 1:**
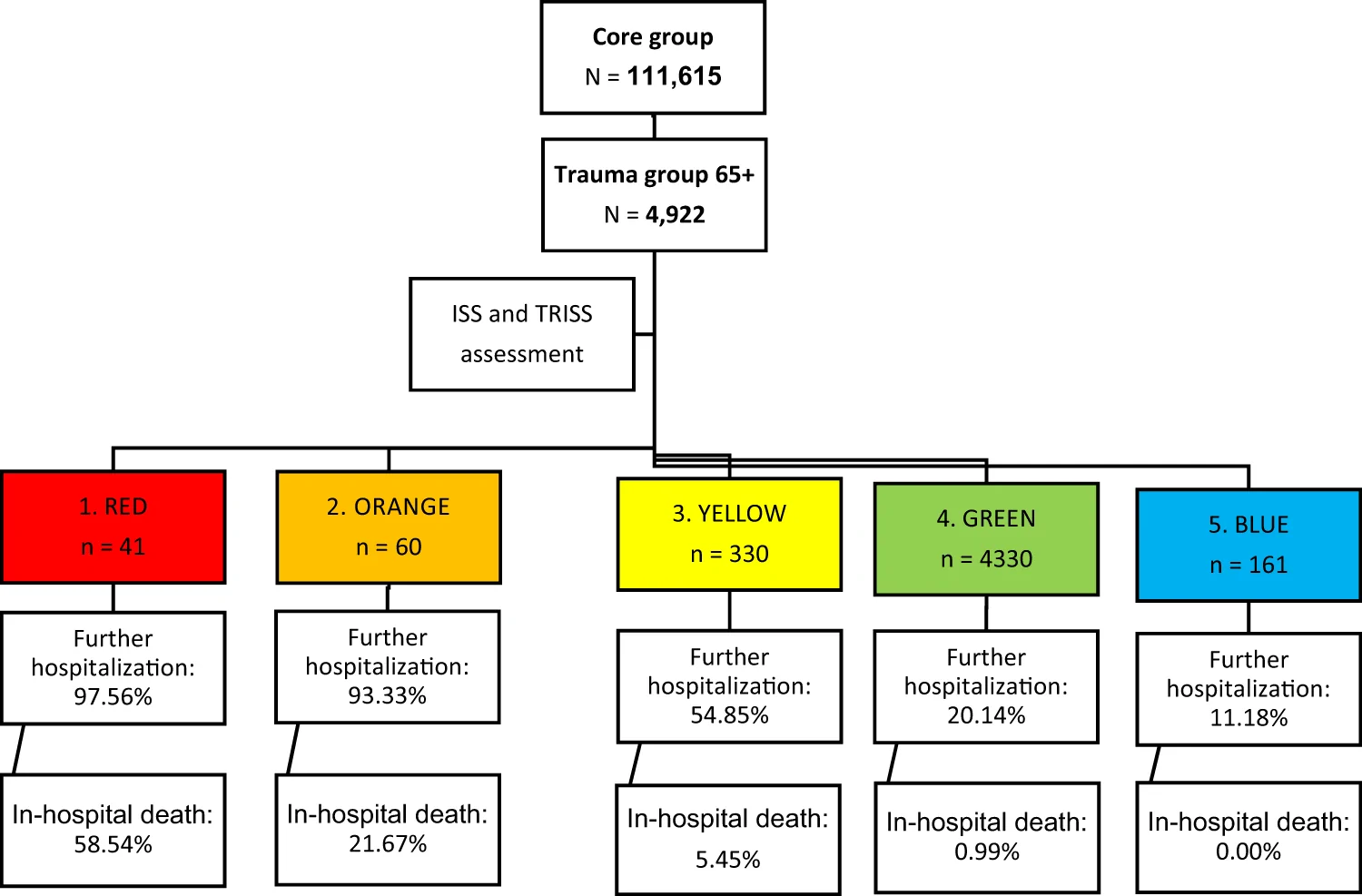
Flowchart illustrating patient selection and study cohort characteristics

During the study period, 111,615 ED admissions were recorded. Among these, 4,936 patients aged 65 years and older admitted following trauma were identified based on ICD-10 diagnostic codes. Fourteen cases were excluded due to missing triage score data, and no other exclusions were necessary. Consequently, the final study cohort comprised 4,922 patients, for whom ISS and TRISS were calculated and triage priority levels were analyzed. Patient selection and study cohort characteristics are shown in Fig. 1.

*The core group* had a mean age of 48.9 years (SD: 21.1), with women accounting for 49.8% and men for 50.2%.

General statistics for *the trauma group 65 +* are presented in Table 1.

**Table 1:** General characteristics of *the trauma group 65+*

Group	*n*	Age M (SD)	Sex (%) F/M	In-hospital death (%) F/M	Further hospitalization (%)	In-hospital survival (%)	ISS Me [Q1–Q3]	TRISS Me [Q1–Q3]
**Trauma group 65+**	4922	77.1 (8.5)	65.4/34.6	1.0/1.0	22.0	98.0	1.00 [1–4]	0.98 [0.98–0.98]

F: female, M: male.

General statistics according to individual triage categories are shown in Table 2

**Table 2:** General statistics according to triage categories

TRIAGE group	*n* (%)	Age M (SD)	Sex (%) F/M	Further hospitalization^&^	In-hospital survival^&^	ISS Me [Q1-Q3]	TRISS Me [Q1-Q3]
1. **RED**	41 (0.8)	73.7 (7.5)	80.5/19.5	97.6	41.5	25 [16–34]	0.25 [0.07– 0.75]
2. **ORANGE**	60 (1.2)	76.5 (8.0)	43.3/56.7	93.3	78.3	16 [9–21]	0.94 [0.74–0.97]
3. **YELLOW**	330 (6.7)	77.8 (9.2)	58.8/41.2	54.8	94.5	4 [1–9]	0.98 [0.97–0.98]
4. **GREEN**	4330 (88.0)	77.1 (8.5)	67.0/33.0	20.1	99.0	1 [1–4]	0.98 [0.98–0.98]
5. **BLUE**	161 (3.3)	75.0 (7.5)	56.5/43.5	11.2	100	1 [1–4]	0.98 [0.98–0.98]

^&^% in triage group; F: female, M: male.

Priority *1. RED* included the smallest proportion of patients (0.83%). In this category, injuries were most frequently located in the head or neck, chest, and external regions, accounting for 73.8% of all cases. The most severe injuries, according to the AIS index, occurred in the head or neck and chest regions (median AIS: 4 for both).

Priority *2. ORANGE* showed that the majority of injuries were located in the head or neck and external regions, accounting for 64.7% of cases. The most severe injuries in this category, based on the AIS index, were observed in the head or neck, chest, and abdominal or pelvic contents regions (median AIS: 3 for each).

Priority *3. YELLOW* had injuries most often located in the external and extremities or pelvic girdle regions, accounting for 68.5% of cases. The most severe injuries, according to the AIS index, were found in the head or neck, chest, and extremities or pelvic girdle regions (median AIS: 3 for each).

Priority *4. GREEN* included injuries predominantly in the external and extremities or pelvic girdle regions, accounting for 87.9% of cases. The most severe injuries in this group, based on the AIS index, were found in the head or neck region (median AIS: 3).

In Priority *5. BLUE*, injuries were mainly located in the external and extremities or pelvic girdle regions, accounting for 85.2% of cases. Among patients aged 65+, the most severe injuries in this category, according to the AIS index, occurred in the head or neck region (median AIS: 3).

**Fig. 2:**
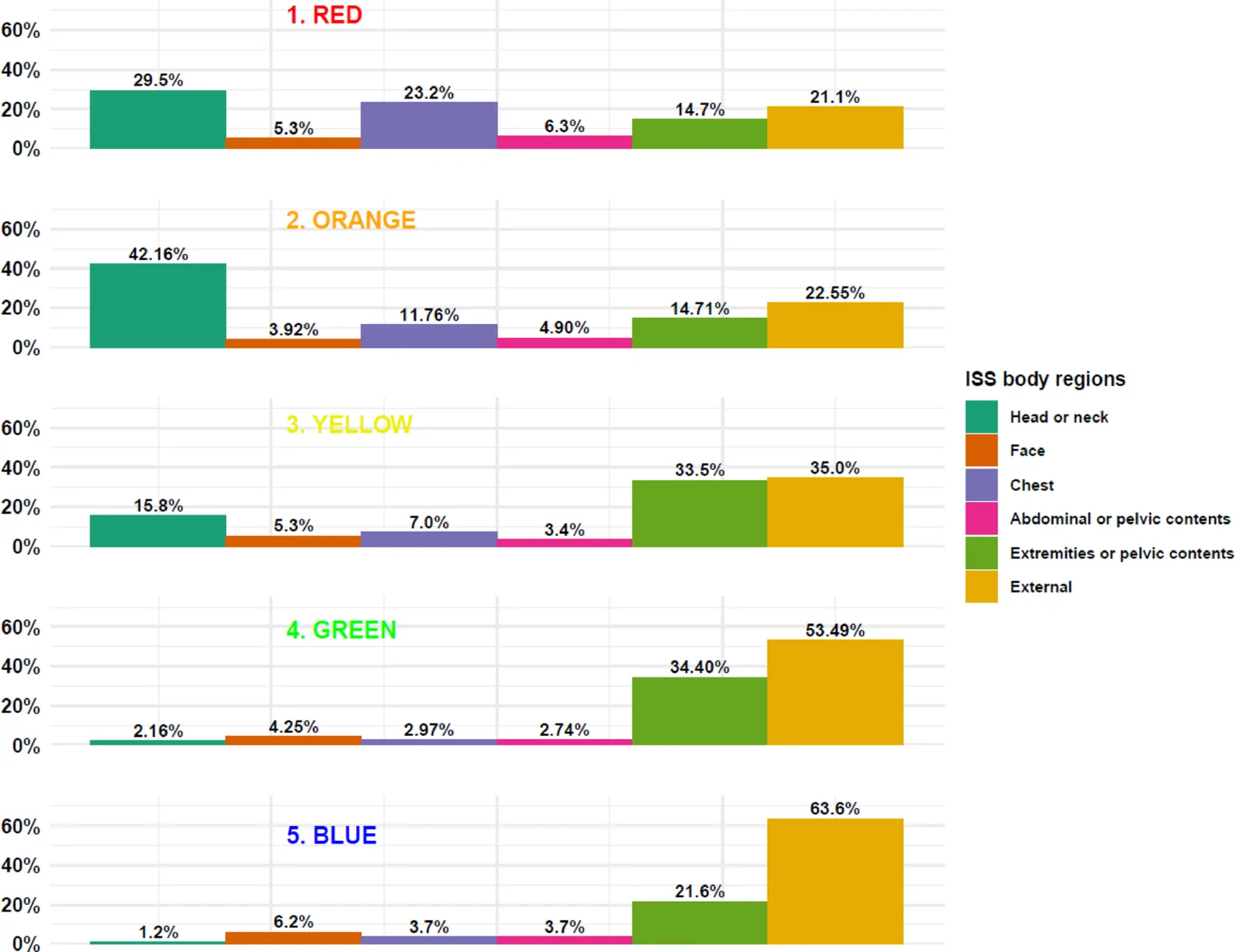
Distribution of injuries across ISS regions by triage priority groups

The distribution of injuries across ISS regions within triage priorities is illustrated in Fig. 2.

General statistics for the analysis of triage groups according to urgency – that is, high-risk priorities (1 + 2) and lower-risk priorities [3–5] – are presented in Table 3.

**Table 3:**
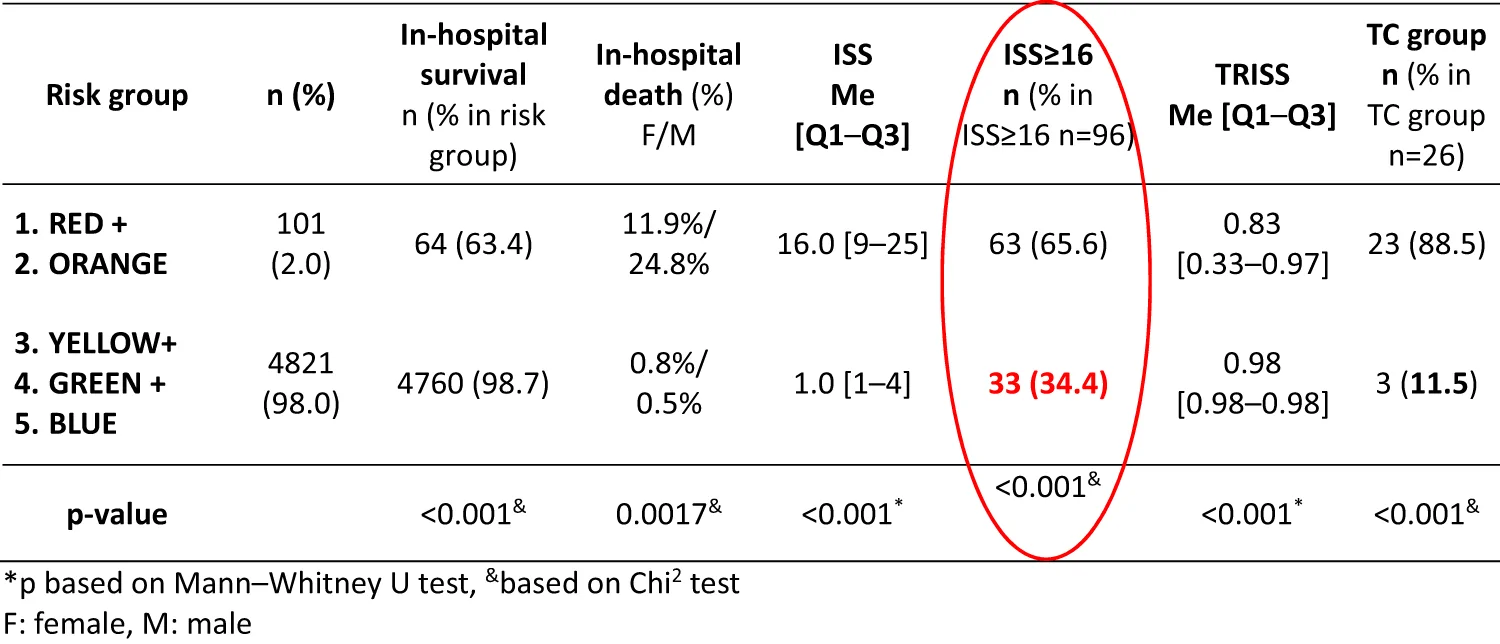
Comparison of urgency groups

Undertriage occurred in 34.4% of severely injured patients, while overtriage was observed in 37.6%.

A multivariable logistic regression model was used to identify characteristics associated with undertriage (Table 4).

**Table 4:** Logistic multivariable model for undertriage

Variable	OR (95% CI)	*p*-value
**Age**,** per 1 year**	1.07 (1.01; 1.13)	0.030
**Sex**,** male vs. female**	2.35 (0.83; 6.68)	0.107
**In-hospital death**,** yes vs. no**	0.75 (0.17; 3.27)	0.705
**TRISS**,** per 0.1 unit**	25.55 (8.03; 81.33)	< 0.001
**ISS**,** per 1 unit**	1.93 (1.67; 2.24)	< 0.001

In the multivariable logistic regression model, older age, higher TRISS, and higher ISS were independently associated with greater odds of undertriage. Each 1-year increase in age was associated with a 7% increase in the odds of undertriage (OR = 1.07, 95% CI: 1.01–1.13, *p* = 0.030). Higher TRISS was also significantly associated with undertriage, with each 0.1-point increase corresponding to markedly higher odds (OR = 25.55, 95% CI: 8.03–81.33, *p* < 0.001). Similarly, each 1-point increase in ISS nearly doubled the odds of undertriage (OR = 1.93, 95% CI: 1.67–2.24, *p* < 0.001). Male sex and in-hospital death were not significantly associated with undertriage.

Based on the undertriage rate, two subgroups were identified:

- **undertriage group 65+** (*n* = 33; ISS ≥ 16 ; triage: 3–5) – patients who should have been assigned to **high-risk priorities**, but whose injuries were underestimated, placing them in lower-risk categories [3–5].
- **correct triage group 65+** (*n* = 63; ISS ≥ 16; triage: 1 + 2) – patients whose injuries were correctly assessed and assigned to high-risk priorities (1 + 2).

Statistical comparisons between *the undertriage group 65 +* and *correct triage group 65 +* are presented in Table 5.

**Table 5:** Comparison between *under- and correct triage groups 65+*

Group	*n* (%)	Age M (SD)	Sex (%) F/M	Further hospitalization (% in group)	In-hospital survival (% in group)	ISS Me [Q1–Q3]	TRISS Me [Q1–Q3]
**Undertriage group 65+**	33 (34.4)	77.6 (9.01)	36.4/ 63.6	91.0	84.8	18 [17–22]	0.93 [0.89–0.94]
**Correct triage group 65+**	63 (65.6)	73.0 (7.75)	30.2/ 69.8	95.2	54.0	24 [17–26.5]	0.52 [0.19–0.87]
**p-value**		0.140^#^	< 0.001^##^	0.0002^##^	0.0003^##^	0.055^*^	< 0.001^*^

* Mann-Whitney U test, ^#^Student T test ^##^ Chi^2^ test.

In *the undertriage group 65+*, the majority of injuries were located in the head or neck, chest, and external regions, accounting for 66% of all cases. In *the correct triage group 65+*, injuries were most frequently located in the abdominal or pelvic contents and head or neck regions, representing 96% of cases. In both groups, the most severe injuries, according to the AIS, occurred in the head or neck region (median AIS: 4 in both groups).

**Fig. 3:**
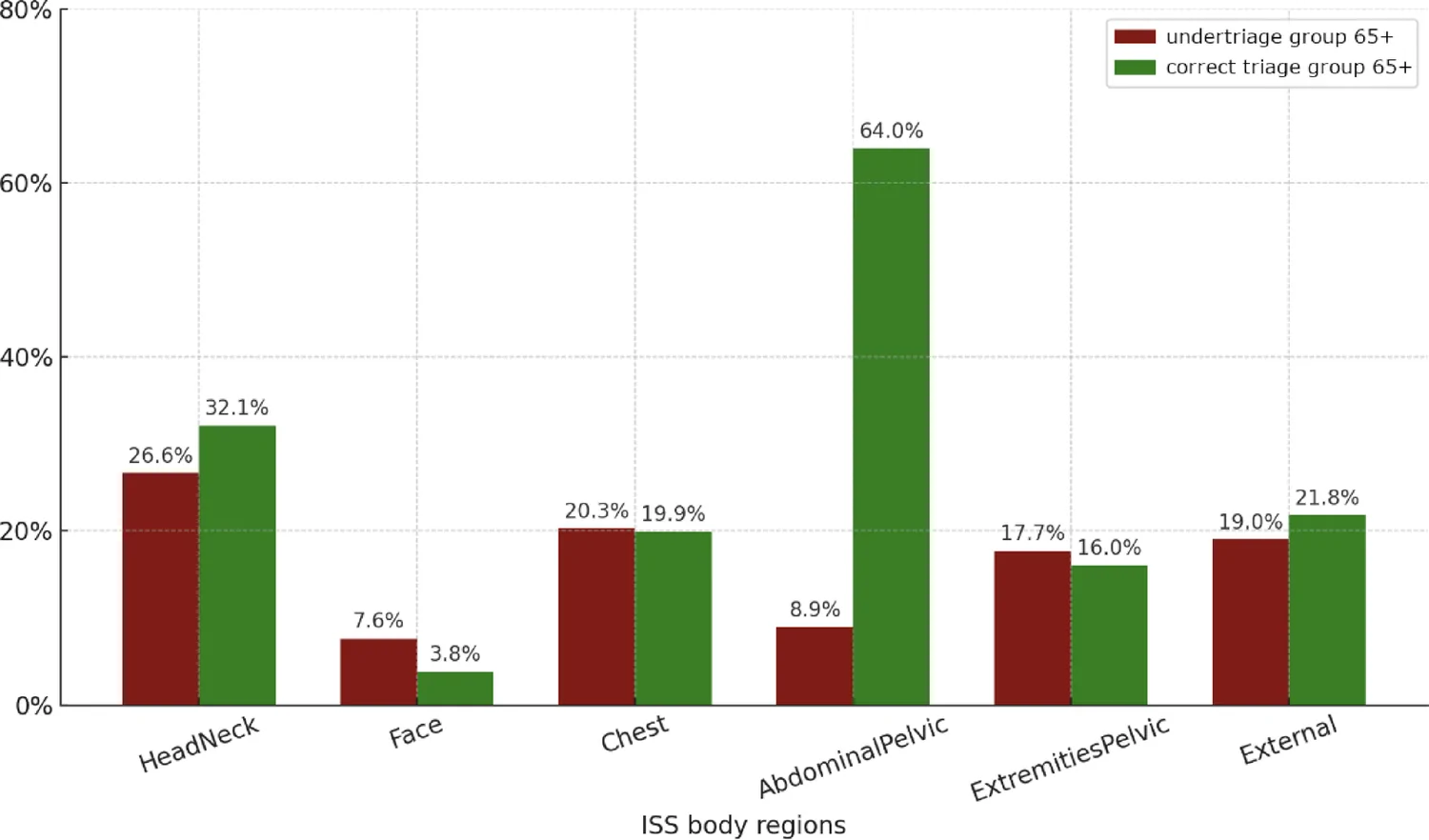
Percentage of injuries by ISS regions in *the undertriage group 65 +* and *correct triage group 65+*

The percentage distribution of injuries by ISS regions in *the undertriage group 65 +* and *correct triage group 65 +* is shown in Fig. 3.

### Analysis of the level of consciousness (GCS) in patients aged 65 + with head and neck injuries

Given the most frequent and severe injuries in *the undertriage group 65 +* involved the head and neck region, an additional analysis of the level of consciousness was performed using the Glasgow Coma Scale (GCS). This post hoc analysis was conducted following the observation of injury distribution patterns. The analysis included patients with head and neck injuries from *the undertriage group 65+* (*n* = 21; 63.3%) and *the correct triage group 65+* (*n* = 50; 79.4%). A statistically significant difference in median GCS values was found between the two groups, with medians of 15 and 5, respectively (Table 6).

In addition, it was found that head and neck injuries accounted for more than half of the overtriaged cases (n = 21; 55%), with a Me GCS of 14 among these patients.

**Table 6:** GCS assessment among patients aged 65 + with head and neck injuries

Group	*n*	GCS Me [Q1–Q3]	*p*-value
**HeadNeck in undertriage group 65+**	21	15 [13–15]	< 0.001
**HeadNeck in correct triage group 65+**	50	5 [3–13]	

p based on Mann-Whitney U test.

## Discussion

The study demonstrated an undertriage rate of 34.4%, which substantially exceeds the < 5% threshold recommended by the ACSCOT [23]. It is important to emphasize that this was a single-center retrospective study conducted in Poland, utilizing manual ISS estimation and no national trauma registry for broader validation; these limitations should be considered when interpreting the findings.

Nevertheless, other studies have also demonstrated the high susceptibility of geriatric trauma patients to undertriage in the ED [1, 20], suggesting that this may be primarily attributable to the limited adaptation of triage algorithms to the specific characteristics of older patients [11].

Age-related pathophysiological changes, including atypical responses to injury or so-called *pseudostability*—that is, apparent clinical stability resulting from an elevated shock threshold [7]—combined with multimorbidity and polypharmacy, particularly anticoagulant therapy [1, 5], complicate accurate injury assessment. In addition, social factors, such as concerns regarding the appropriateness or futility of intensive treatment in older adults, may also influence therapeutic decisions [8–10]. Undertriage at ED admission might likewise reflect unconscious age bias—a tendency to assign lower triage priority in cases of seemingly minor mechanisms, such as falls, because of concerns about ED resource burden and perceived futility of intervention (4).

In geriatric populations, commonly used physiological triage parameters fail to accurately reflect actual injury severity due to physiological changes associated with aging and reduced compensatory reserves [4]. This may mask clinical severity, for example, through the absence of tachycardia despite hypovolemia or by only minimal changes in consciousness despite traumatic brain injury [11]. Such underestimation of injury severity can lead to significant delays in diagnostic and therapeutic management, increasing the risk of complications and mortality.

Together, these factors increase the risk of undertriage, potentially leading to significant, life-threatening delays in diagnosis and intervention or, in extreme cases, to omission of appropriate treatment [11].

In the multivariable analysis, undertriage was associated with increasing injury severity (ISS) and higher predicted survival (TRISS). This finding suggests that, in this cohort, severely injured older patients with apparently favourable physiological profiles may have been vulnerable to underestimation during ED triage. The most severe injuries across all analyzed groups involved the head and neck region (median AIS: 3–4). In priorities *(1) RED* and *(2) ORANGE*, head and neck injuries predominated, with median AIS values of 4 and 3, respectively. In priorities *(3) YELLOW*,* (4) GREEN*, and *(5) BLUE*, the most common injuries occurred in the external and extremities or pelvic girdle regions. However, the most severe injuries in these lower-risk priorities were still located in the head and neck region (median AIS: 3). Injuries of comparable severity were also noted in the chest and extremities or pelvic girdle regions in priority *3. YELLOW*. The median AIS for the head and neck was 4 in both groups – *undertriage* and *correct triage groups 65+.*

These findings align with previous studies reporting the predominance of head, lower extremity, and thoracic injuries among geriatric patients [4, 5]. Previous reports have further highlighted the growing incidence of severe fall-related head injuries, which account for approximately 30% of all injuries in this population [5].

In this study, the median ISS was 18 in *the undertriage group 65 +* and 24 in *the correct triage group 65+.* A significant difference was also observed in the median GCS between patients with head and neck injuries in *the undertriage group 65 +* and *correct triage group 65+* (15 vs. 5), highlighting the limited effectiveness of GCS as a tool for assessing severity of traumatic brain injury in geriatric patients.

Similar findings have been reported by Park et al. (2025), who found that undertriaged geriatric patients were statistically older (77 vs. 73 years), had lower ISS (25 vs. 27), and mostly had head and neck injuries (89% vs. 65%). Additionally, undertriaged patients had higher initial GCS scores (15 vs. 14), which could contribute to misjudgement of clinical severity. For comparison, correctly triaged patients had a higher proportion of severe head and neck injuries (AIS ≥ 3, 89% vs. 65%). Patients with severe injuries to the chest, abdomen, pelvis, and extremities were also typically classified appropriately [7]. Consistent with this observation, injuries to the abdominal or pelvic contents were most frequently observed in *the correct triage group 65 +* in the present study.

Analysis revealed that the level of consciousness assessed by the GCS, significantly influenced triage decisions among patients with head and neck injuries. In the undertriaged group, the median GCS score was 15, indicating no evident impairment of consciousness, which may have led to an underestimation of clinical severity. This issue is particularly relevant in geriatric patients, in whom age-related brain atrophy and increased intracranial space may allow initially high GCS scores despite progressive hematoma formation, thereby delaying appropriate intervention [4]. In contrast, in *the correct triage group 65+*, head injuries were present in 79.4% of cases, with a median GCS score of 5. Such profound neurological impairment constituted a clear clinical indicator for urgent intervention and naturally resulted in assignment to a high-risk triage priority. Among overtriaged patients, 55% had head and neck injuries with a median GCS score of 14. These discrepancies suggest the limited sensitivity of standard triage tools in geriatric populations, particularly with regard to central nervous system assessment. The GCS scale, when used as a stand-alone decision-making tool, may be insufficient.

Conversely, the overtriage rate was 37.6%, exceeding the ACSCOT-recommended range 25–35% [23]. In another study, which reported an overtriage rate of 40%, the authors emphasized that although overtriage may theoretically improve injury detection, it can also lead to excessive resource utilization and system overload [1], potentially delaying care for critically ill patients [11]. High overtriage rates may additionally reduce staff vigilance and increase procedural costs without clear clinical benefit [1].

Detailed analysis showed that 55% of overtriaged patients sustained head and neck injuries with a median GCS score of 14. This may reflect a cautious clinical approach driven by awareness of potential intracranial complications, even after low-energy trauma [24]. Severe head and neck injuries predominated in both the overtriaged (55%) and undertriaged (63.3%) groups. These observations may indicate limitations of current triage criteria for cranio-cerebral trauma, particularly traumatic brain injury, in the geriatric population.

Overall, in-hospital survival in the study cohort was 98.0%. Survival was higher in the lower-risk triage priorities [3–5], reaching 98.7% (median ISS: 1), but declined markedly in high-risk priorities (1 + 2), where it was 63.4% (median ISS: 16). The TRISS score indicated similar survival (98%) in lower-risk priorities [3–5], but overestimated survival in high-risk priorities (1 + 2), predicting 83%. This finding suggests that score accuracy may be limited in cases of severe injury, although it is generally considered a reliable trauma scoring system for predicting mortality in geriatric patients (22).

In addition, in the analyzed group of patients aged 65 and older admitted to the ED due to trauma, women predominated (65.4%). This female predominance was also observed across most triage categories, except for the priority *2. ORANGE*, where men constituted the majority (56.7%). In contrast, among patients with severe injuries (ISS ≥ 16), men predominated in both *undertriage* and *correct triage 65 + groups* (63.6% and 69.8%, respectively). Some studies suggest that women are more susceptible to undertriage than men, likely due to a higher prevalence of osteoporosis and increased vulnerability to severe injuries from low-energy mechanisms, such as falls [7, 21]. Conversely, older men have been reported to experience significantly higher post-traumatic mortality compared to women [4].

In the present study, in-hospital mortality did not differ significantly between sexes (1% for both men and women). However, in the high-risk triage priorities (1 + 2), mortality was twice as high among men (24.8%). Literature emphasizes that although sex-related differences may influence injury epidemiology, they do not directly impact therapy [5].

The initial assessment of geriatric patients represents a significant clinical challenge. The ESI manual notes that the absence of typical compensatory responses, due to aging and chronic pharmacotherapy, requires vital signs to be interpreted in the context of medication use before assigning an ESI level [17]. High sensitivity to subtle signs of severe injury is particularly important in the context of hypovolemic shock, recognized as one of the leading preventable causes of death in geriatric trauma patients. In hypovolemia, tachycardia and hypotension are key clinical indicators. However, aging and pharmacotherapy may blunt these responses, falsely suggest hemodynamic stability, and thereby contribute to undertriage [4, 25].

Most available studies focus on prehospital criteria for triage to TCs and trauma team activation (TTA), particularly on modifying parameters that can also be applied during ED triage [26–28]. Various modifications have been proposed, including raising the SBP threshold to 110 mmHg as a more appropriate indicator of post-traumatic hypotension in geriatric patients [4]. This value is associated with increased mortality, especially among older adults with traumatic brain injury (TBI) [1]. The shock index (HR/SBP ratio) has also been suggested as a potentially better predictor of mortality in this population. Other recommendations include lowering the GCS < 13 or 14 and correlating it with the mechanism of head trauma. Hospitals have additional time and resources, which allow for a more accurate clinical assessment. Although the complexity of triage algorithms has a limited impact on clinical outcomes, studies indicate their positive effect on reduced mortality and shorter intervention times [1]. Hospital triage protocols may include point-of-care (POC) diagnostics, activation of the trauma team (TT) within the trauma care system, referral of high-risk patients to the ICU, and early involvement of geriatric specialists within multidisciplinary trauma care teams [1].

One study described a collaborative ED – trauma team model that incorporated early assessment of occult hypoperfusion (base deficit, BE< − 2), which was associated with increased mortality and complication risk among geriatric trauma patients [1]. Technological advances and wider access to POC diagnostic tools enable implementation of more sophisticated triage algorithms tailored to the needs of geriatric patients. The potential use of artificial intelligence to enhance triage accuracy is also increasingly being considered [1].

Effective management of geriatric trauma patients requires a multidisciplinary approach, and evidence suggests that treatment in trauma centers (TCs) is associated with improved in-hospital survival [2, 4, 7]. Patients may be referred to TCs directly from prehospital services or via inter-hospital transfers. Regardless of the admission pathway, they undergo triage assessment in the ED. The 2023 ACSCOT guidelines recommend modified qualification criteria for older patients, incorporating injury mechanism and adjusted physiological parameters [1]. Treatment in a TC is advised for severe injuries, defined as an ISS ≥ 16 [4].

In systems where age is not an independent TC admission criterion, identification of severely injured patients may occur already at the ED through rapid physical assessment or laboratory testing. This allows for trauma team activation within 30 min [1].

In this analysis, 11.5% of patients who met TC admission criteria were assigned lower triage priorities [3–5]. Similar findings have been reported in another study of patients aged ≥ 70 years, in which only 25% met trauma team activation (TTA) criteria, although 65% had an ISS > 15 [1]. These observations highlight the limitations of the current national criteria for admission to trauma centers (TCs).

Notably, these criteria have also been regarded as overly restrictive in other national studies, particularly with regard to geriatric patients. They fail to account for the specific characteristics of older adults, which may contribute to undertriage [26].

At the same time, several authors have emphasized that triage criteria with high sensitivity must also retain adequate specificity to ensure both clinical and economic feasibility. Otherwise, the practical implementation of such protocols within trauma systems becomes unrealistic [1]. For example, in the study by Maughan et al., modification of triage criteria increased the proportion of severely injured patients transferred to TCs (from 24% to 37%) but also resulted in a significant increase in healthcare costs [1].

Triage in the geriatric population remains particularly complex, and no standardized system ensuring its effective implementation has yet been established. Existing geriatric triage approaches demonstrate substantial variability with respect to qualification criteria and age thresholds, while their clinical effectiveness and cost efficiency remain inconclusive [1]. Future research should therefore focus on refining geriatric-specific triage criteria to achieve an optimal balance between sensitivity and specificity—an essential prerequisite for improving quality of care, optimizing resource utilization, and enhancing survival outcomes among geriatric trauma patients.

This study has several important limitations that should be considered when interpreting the results. First, the analysis was retrospective and based on medical documentation, which may have involved incomplete or missing clinical data, such as mechanism of injury, comorbidities, medication use, or pre-injury functional status. Second, as no national trauma registry currently exists in Poland, international comparisons and broader population-level validation remain limited. Consequently, ISS values were estimated manually, increasing the risk of subjectivity and potential classification errors. It should also be emphasized that the ISS is a retrospective measure of injury severity based on comprehensive diagnostic data, while ED triage is a real-time prioritization process relying on initial presentation. Additionally, the study included only patients admitted to a single emergency department and was conducted using a triage system based on the Emergency Severity Index (ESI), which was subsequently adopted as a nationwide standard; however, no detailed formal validation procedures have been defined. This limits the generalizability of the findings to the entire geriatric population. Finally, the study did not include long-term outcomes, such as survival, quality of life, or functional recovery, which are key indicators of treatment effectiveness in geriatric trauma care.

## Conclusion

Geriatric patients constitute a particularly challenging population in trauma care, with a high observed rate of undertriage (34.4%) in the present cohort. Head and neck injuries were the most severe across all triage priority levels, suggesting that current ED triage protocols may insufficiently capture injury severity in older adults, particularly in cases of head trauma. In this cohort, commonly used physiological triage parameters, including GCS, were associated with limitations in identifying patients with more severe injuries.

Taken together, these findings support the need for further research on geriatric-specific triage approaches that account for age-related physiological changes and other factors potentially influencing triage accuracy and the management of older trauma patients.

## Data Availability

No datasets were generated or analysed during the current study.
